# An extrapancreatic solid-pseudopapillary neoplasm in the greater omentum

**DOI:** 10.1259/bjrcr.20170008

**Published:** 2017-03-10

**Authors:** Akane Yoshikawa, Yasuji Ryu, Harumi Takata, Yoshihide Asaumi, Mitsuaki Sakatoku, Shintaro Terahata

**Affiliations:** ^1^Department of Radiology, Tonami General Hospital, Tonami, Japan; ^2^Department of Surgery, Tonami General Hospital, Tonami, Japan; ^3^Department of Pathology, Tonami General Hospital, Tonami, Japan

## Abstract

A solid pseudopapillary neoplasm (SPN) is an uncommon pancreatic tumour that usually occurs in young women. Tumours outside the pancreas (e.g. in the ovary, retroperitoneum or omentum) are rare. We report a case of an SPN arising from the greater omentum in a 78-year-old male who presented with a month-long history of abdominal pain and a palpable abdominal mass. Laboratory data showed inflammation and anaemia. CT and magnetic resonance imaging revealed a well-defined encapsulated mass measuring 18 cm in the upper right abdomen. The tumour was completely removed via surgery, and pathologic examination confirmed a diagnosis of an SPN in the greater omentum.

## Clinical presentation

A 78-year-old male presented at a local hospital with a month-long history of abdominal pain and a palpable abdominal mass. Non-contrast-enhanced CT detected an intraperitoneal mass, and he visited our hospital for further examination. Laboratory data showed inflammation and anaemia. Carcinoembryonic antigen and carbohydrate antigen 19-9 levels were normal.

## Investigation/imaging findings

**Figure 1.**
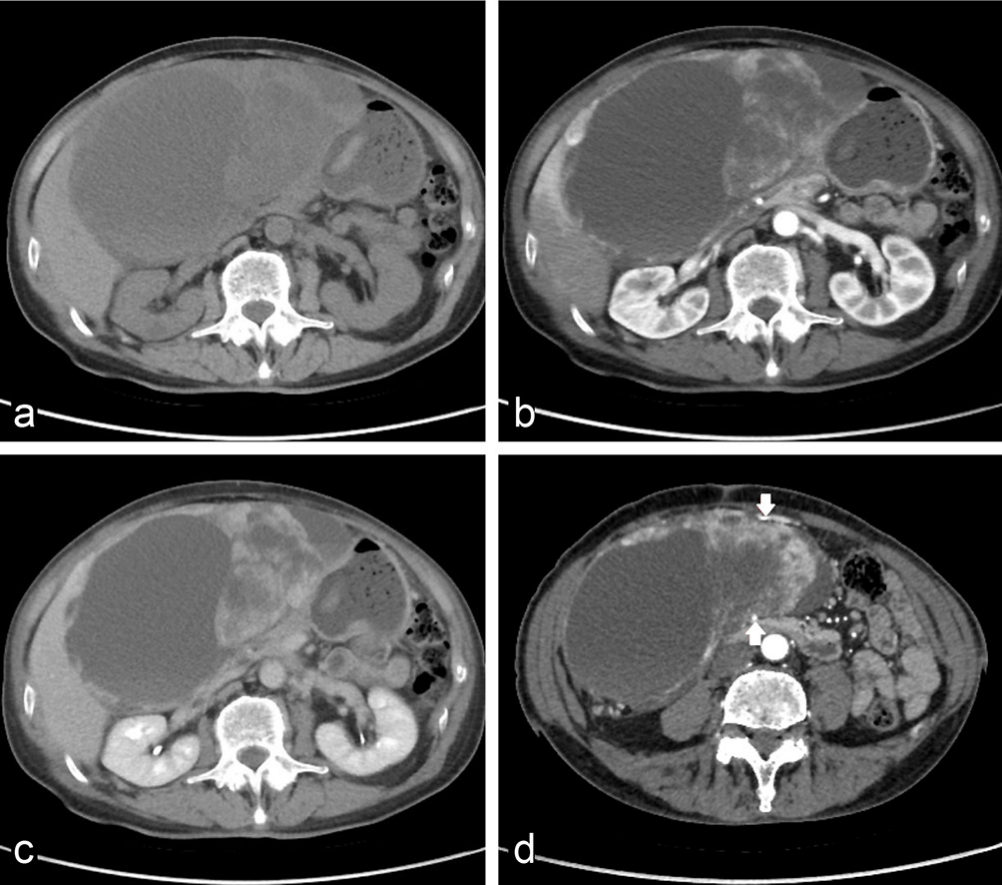
CT scans show a well-encapsulated heterogeneous mass in the upper abdominal region. The mass was surrounded by the liver, stomach, transverse colon and close to the gallbladder. (a–c) Contrast-enhanced CT scans show early peripheral heterogeneous enhancement of the solid portion of the mass with progressive fill-in. (d) The main blood supply originated in the branches of the right gastro-omental artery (arrow).

CT revealed a well-defined intraperitoneal encapsulated solid mass with cystic components but no calcification. The mass was 18 cm in diameter, with the solid portion at the periphery. It was located in the upper abdominal region, surrounded by the liver, stomach, transverse colon, and close to the gallbladder (Figure 1). Its main blood supply originated in the branches of the right gastro-omental artery. No abnormalities were observed in the pancreas.

**Figure 2.**
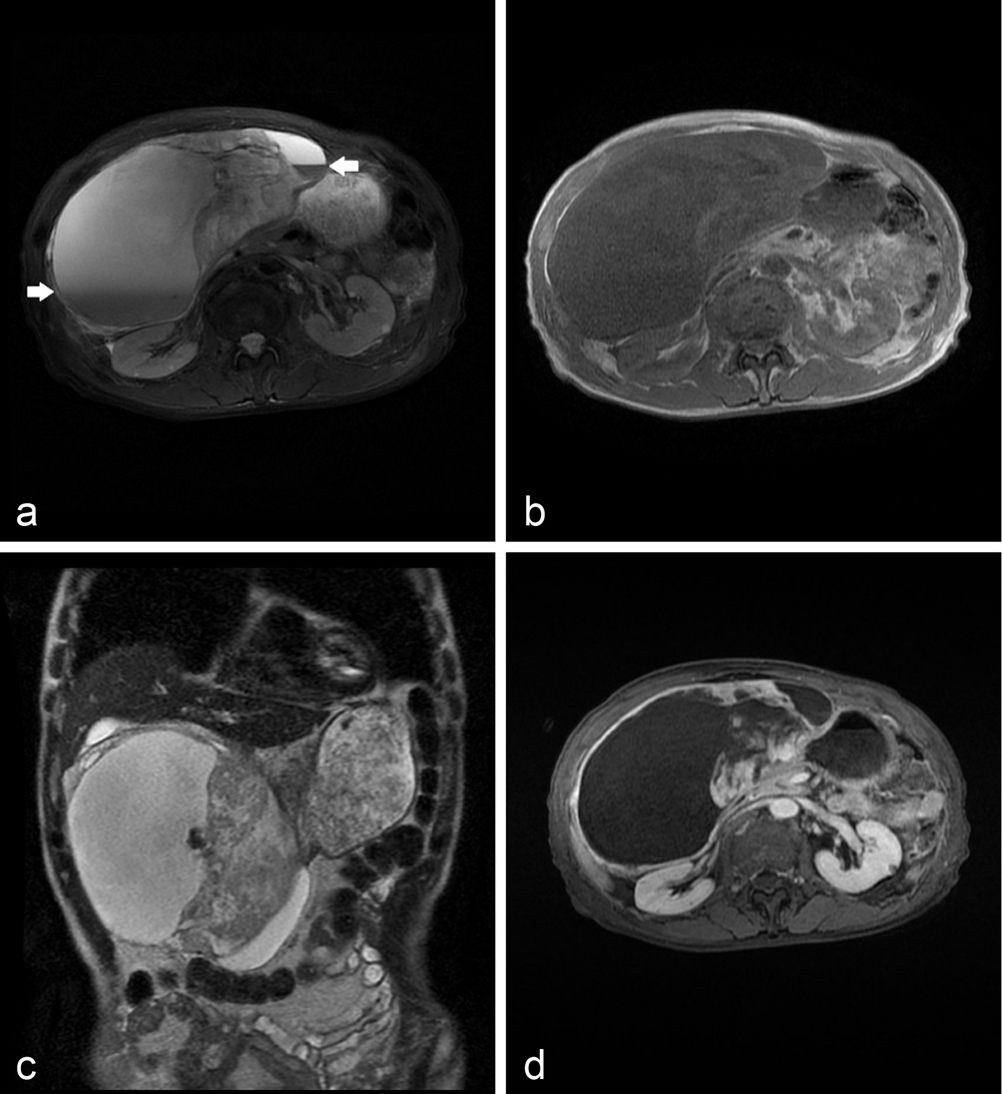
(a) A *T*_2_ weighed image shows fluid–fluid levels within the mass, indicating haemorrhage (arrow). (b) A *T*_1_ weighed gradient-echo image shows a heterogeneous mass. (c) A heavy *T*_2_ weighted image shows a rim of low signal intensity around the mass. (d) A delayed magnetic resonance image shows heterogeneous enhancement of the solid portion of the mass.

MRI revealed a well-defined lesion with a mix of high and low signal intensity on *T*_1_ and *T*_2_ weighted images. On the *T*_2_ weighted image, the mass was rimmed by low signal intensity and contained fluid–fluid levels. Gadolinium-enhanced dynamic MRI showed early peripheral heterogeneous enhancement of the solid portion with progressive fill-in (Figure 2). The suggested diagnosis was a gastrointestinal stromal tumour (GIST) in the greater omentum or stomach, and open surgery was performed.

## Treatment/outcome/follow-up

During surgery, a well-defined mass was found in the upper abdominal region.

The tumour adhered to the stomach and gallbladder, but was clearly separate from the pancreas and transverse colon. Tumourectomy with cholecystectomy and distal gastrectomy was performed.

**Figure 3.**
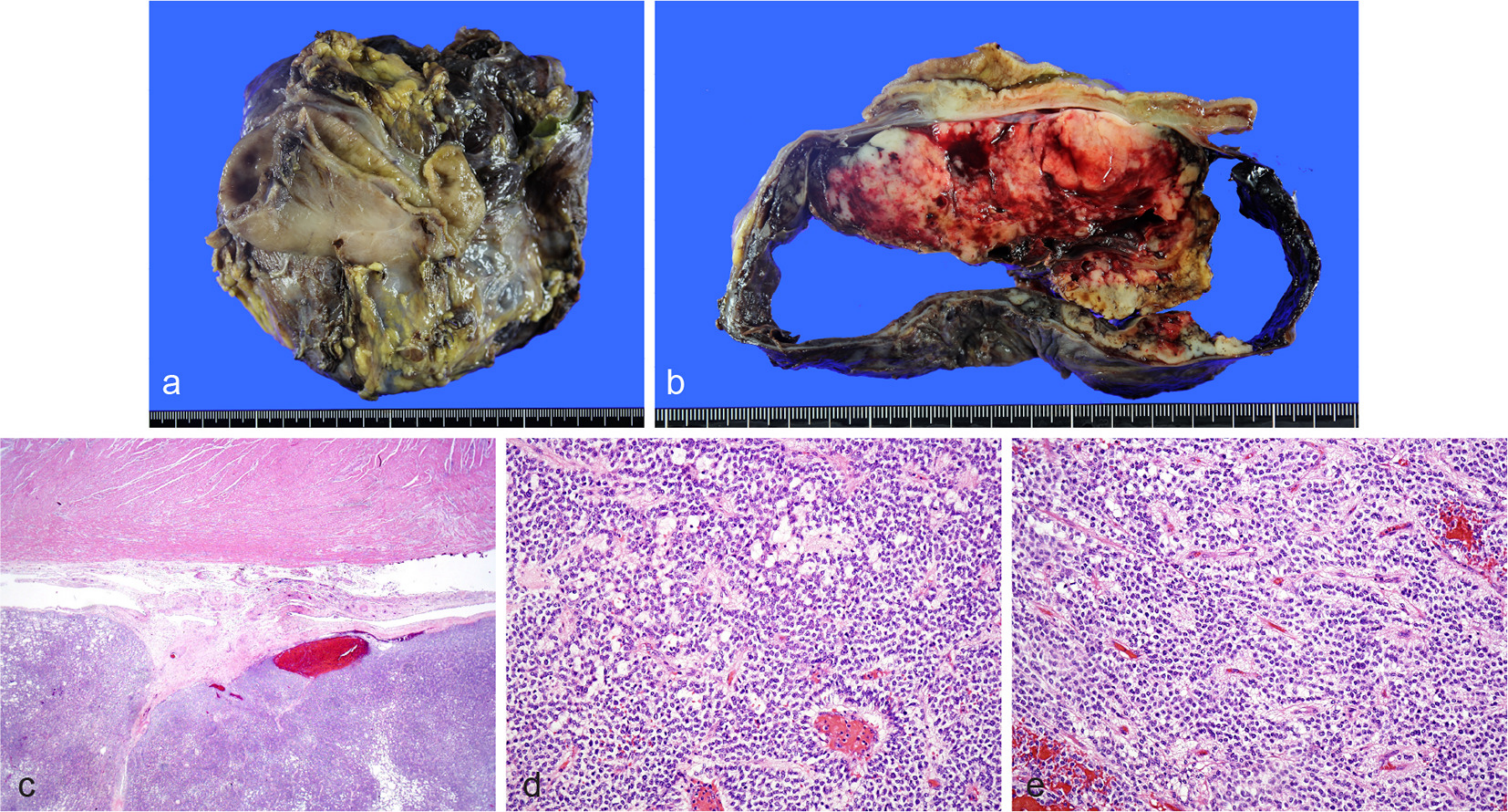
(a, b) Grossly, the tumour was solid and cystic, with areas of necrosis and haemorrhage. The stomach and gallbladder were attached to the mass. (c) Microscopically, the tumour had a fibrous capsule, which was separated from the stomach, and part of the tumour was in the gastrocolic ligament. Haematoxylin and eosin, ×20. (d, e) The tumour was composed of cells arranged in solid sheets and microcysts with a fibrovascular core. The tumour cells had round or oval nuclei and eosinophilic or lightly coloured cytoplasm. Haematoxylin and eosin, ×100.

Macroscopically, the resected tumour was a circumscribed mass measuring 16 cm. The external surface was smooth, and the cut surfaces of the tumour showed interspersed cystic, haemorrhagic and necrotic spaces (Figure 3a,b). The tumour was attached to the stomach and gallbladder and was partly in the gastrocolic ligament.

Microscopically, the tumour had a fibrous capsule. The surgical sample contained normal stomach and gallbladder tissue, which were separate from the fibrous capsule (Figure 3c). The tumour was composed of cells arranged in solid sheets and microcysts with a fibrovascular core. The tumour cells had round or oval nuclei and eosinophilic or lightly coloured cytoplasm (Figure 3d,e). Regional cystic degeneration, haemorrhage and necrosis were observed. Extensive sampling found no ectopic pancreatic tissue within or adjacent to the tumour.

Immunohistochemically, the tumour cells were positive for vimentin, CD56, DOG1 (discovered on GIST-1), cytoplasmic β-catenin, and neuron-specific enolase and negative for AE1/AE3, CAM5.2, E-cadherin, c-kit, desmin, CD34, CD10, progesterone receptors, cyclin D1 and CK19.

**Figure 4.**
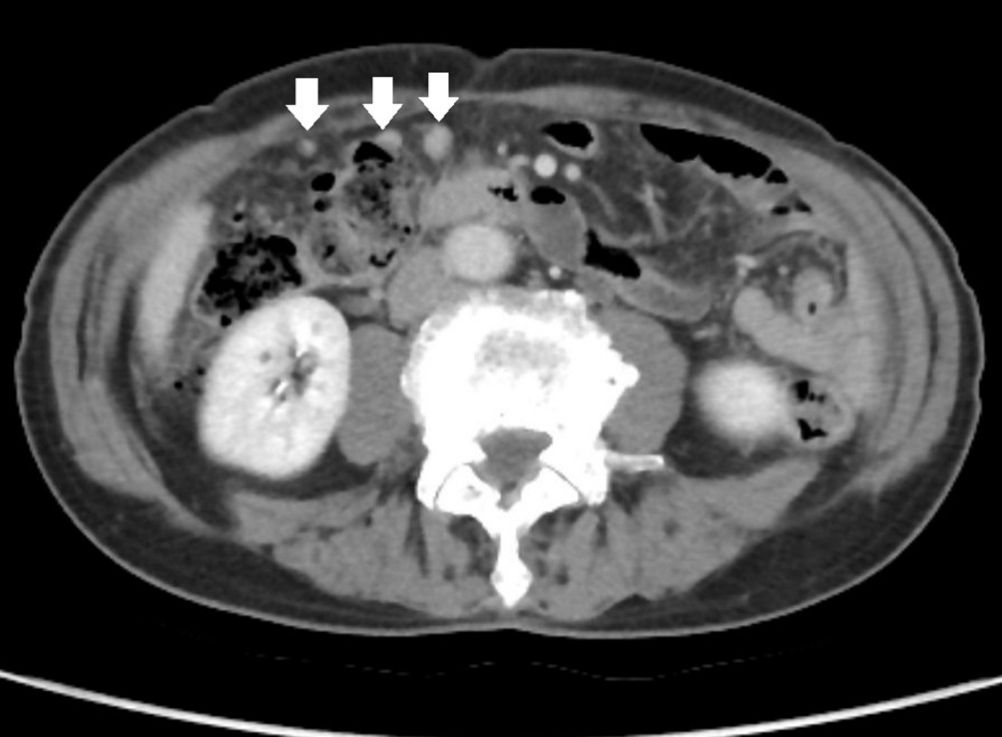
A CT scan 10 months after surgery shows peritoneal metastasis (arrow).

Based on the morphological and immunohistochemical findings and the absence of a pancreatic mass, the final pathological diagnosis was an SPN arising from the greater omentum. Ten months after surgery, peritoneal metastasis was detected via CT (Figure 4).

## Discussion

SPNs are relatively rare tumours usually found in the body or tail of the pancreas. They typically occur in young women in the second to fourth decade of life. Before being officially designated as SPNs of the pancreas by the World Health Organization in 2000, they were referred to as solid and cystic tumours, solid and papillary epithelial neoplasms, papillary cystic tumours and Frantz’s tumours.^1,2^

Although more and more SPN cases have been reported in recent years, primary SPNs outside the pancreas are exceedingly rare. To the best of our knowledge, there are only 19 cases of extrapancreatic SPNs in the English literature.^3–18^ In these cases, the primary site was the mesocolon, ovary, greater omentum, retroperitoneum or liver. Some SPNs are thought to arise from an ectopic pancreas, while others lack ectopic pancreatic tissue. An ectopic pancreas is detected via autopsy at a reported frequency of 0.6–13.7%. It occurs in the upper gastrointestinal tract, mainly in the stomach, duodenum, and jejunum, but has also been observed in a Meckel’s diverticulum and the ileum, gallbladder and omentum.^19^

In the present case, the tumour was located in the upper abdominal region. We believe that it originated in the greater omentum for two reasons: the fibrous portion of the tumour was separated from the stomach and gallbladder, and part of the tumour was in the gastrocolic ligament as determined via microscopic analysis. As further support, CT revealed that the tumour’s main blood supply originated in the branches of the right gastro-omental artery. There was no definitive evidence of an ectopic pancreas origin.

The aetiology of SPN development is not well established. Although the immunohistological features of SPNs suggest an epithelial origin, Kosmahl et al. suggest that SPNs arise from genital ridge-related cells that were incorporated into the pancreas during organogenesis.^20^ This hypothesis might explain why the SPNs in the present case and previous cases lacked ectopic pancreatic tissue.

SPNs of the pancreas have distinctive pathological features. Microscopically, they display two distinct types of growth patterns, namely, solid and pseudopapillary. The tumour cells are characterized by round to oval nuclei and abundant pale to eosinophilic cytoplasm. On immunohistochemistry, they typically express vimentin, CD10 and nuclear and cytoplasmic β-catenin, but not chromogranin A.^21^ However, in the present case, some tumour cells were negative for CD10 and nuclear β-catenin. Although this immunohistochemical pattern was not typical, the appearance of the tumour tissue stained with haematoxylin and eosin was consistent with that of an SPN, hence leading to our final diagnosis of an SPN arising from the greater omentum.

The classic CT feature of pancreatic SPNs is a large well-encapsulated mass with varying amounts of solid and cystic components that reflect the degree of haemorrhagic degeneration. Calcification may exist at the periphery of the mass. Contrast-enhanced dynamic CT scans show early peripheral heterogeneous enhancement of the solid portion of the mass with progressive fill-in. MRI typically shows a well-defined heterogeneous mass with thick fibrous capsule that appears as a discontinuous rim of low signal intensity on *T*_2_ weighted images. Areas of high signal intensity on *T*_1_ weighted images and fluid-fluid levels within the mass on *T*_2_ weighted images can help identify blood products.^2^ The imaging features of the extrapancreatic SPNs in the present case and previous cases were similar to those of conventional pancreatic SPNs.

Pancreatic SPNs have a limited malignant potential, and hence tend to have a favourable prognosis with little local recurrence in studies with long-term follow-ups. Although extrapancreatic SPNs are similar in these respects, careful follow-up is needed because some SPNs can metastasize as is the present case.

## Learning points

1. Primary SPNs occurring outside the pancreas are exceedingly rare.
2. The imaging features of extrapancreatic SPNs in the present case and previous cases are similar to those of conventional pancreatic SPNs.
3. Although preoperative diagnosis of an extrapancreatic SPN remains challenging, the presence of an encapsulated solid and cystic mass with calcification and haemorrhage is at least suggestive of this diagnosis.

## Consent

Informed consent was obtained from the patient after explaining the publication process and use of images.
